# Predictive Association of Smoking with Depressive Symptoms: a Longitudinal Study of Adolescent Twins

**DOI:** 10.1007/s11121-019-01020-6

**Published:** 2019-05-08

**Authors:** Anu Ranjit, Jadwiga Buchwald, Antti Latvala, Kauko Heikkilä, Annamari Tuulio-Henriksson, Richard J. Rose, Jaakko Kaprio, Tellervo Korhonen

**Affiliations:** 1 Department of Public Health, University of Helsinki, Helsinki, Finland; 2 Institute for Molecular Medicine Finland, University of Helsinki, Helsinki, Finland; 3 Department of Psychology and Logopedics, University of Helsinki, Helsinki, Finland; 4 Department of Psychological and Brain Sciences, Indiana University, Bloomington, IN USA

**Keywords:** Depression, Depressive symptoms, Cigarette smoking, Adolescence, Twin study

## Abstract

Longitudinal, genetically informative studies of the association between cigarette smoking and depressive symptoms among adolescents are limited. We examined the longitudinal association of cigarette smoking with subsequent depressive symptoms during adolescence in a Finnish twin cohort. We used prospective data from the population-based FinnTwin12 study (maximum *N* = 4152 individuals, 1910 twin pairs). Current smoking status and a number of lifetime cigarettes smoked were assessed at the age of 14 and depressive symptoms at the age of 17. Negative binomial regression was conducted to model the association between smoking behavior and subsequent depressive symptoms among individuals, and within-pair analyses were conducted to control for unmeasured familial confounding. Analyses were adjusted for age, sex, school grades, drinking alcohol to intoxication, health status, family structure, parental education, and smoking, as well as for pre-existing depressiveness. The results of the individual-level analyses showed that cigarette smoking at the age of 14 predicted depressive symptoms at the age of 17. Compared to never smokers, those who had smoked over 50 cigarettes (incidence rate ratio, IRR = 1.43, 95% CI 1.28–1.60) and regular smokers (IRR = 1.46, 95% CI 1.32–1.62) had higher depression scores. The associations were attenuated when adjusted for measured covariates and further reduced in within-pair analyses. In the within-pair results, the estimates were lower within monozygotic (MZ) pairs compared to dizygotic (DZ) pairs, suggesting that shared genetic factors contribute to the associations observed in individual-based analyses. Thus, we conclude that cigarette smoking is associated with subsequent depressive symptoms during adolescence, but the association is not independent of measured confounding factors and shared genetic influences.

**Electronic supplementary material:**

The online version of this article (10.1007/s11121-019-01020-6) contains supplementary material, which is available to authorized users.

## Background

Cigarette smoking is one of the most important causes of mortality and morbidity, being responsible for several health consequences such as lung cancer and chronic obstructive pulmonary disease (Drope et al. 2018). Smoking initiation can occur early, even before the age of 10 (Global Youth Tabacco Survey Collaborative Group 2002). In a recent European survey, more than one in five had smoked cigarettes by the age of 13 (ESPAD 2016). Exposure to nicotine during adolescence is associated with depressiveness in adulthood (Iniguez et al. 2009). During a depressive episode, a person experiences depressed mood, loss of interest, and reduced energy affecting day-to-day activity for a period of at least 2 weeks (American Psychiatric Association 2013). The prevalence of major depressive disorder experienced by the age of 18 has been estimated at 11.0% (Avenevoli et al. 2015). This is not far from the estimated prevalence among adults (17%) (Kessler et al. 2005). Cigarette smoking and depression cause psychosocial and economic burden both at the individual and societal levels.

Adolescence is undoubtedly a crucial phase in life for the development of both addictive behaviors and depression, and these conditions often exist comorbidly (Royal College of Psychiatrists 2013). Among adults, the association between cigarette smoking and depression is well established, such that smoking exposure is associated with subsequent depression, and also that depression is associated with later smoking behavior (Fluharty et al. 2017). Similar findings have been reported also in adolescents (Chaiton et al. 2009) but the mechanisms underlying the associations remain poorly understood. Hence, more evidence is needed to understand the development of the link between smoking and depression.

Inferring causality between smoking and depressive symptoms in observational studies is nearly impossible. Published molecular genetic studies using the Mendelian randomization approach have not supported causality (Taylor et al. 2014a). However, experimental studies have suggested a possible causal mechanism: chronic exposure to nicotine may lead to neurobiological changes, for example, in the release of dopamine and serotonin which are associated with depressive symptoms (Balfour and Ridley 2000; Rao 2006). Importantly, since smoking is a modifiable risk factor, preventing smoking in the first place and providing support to young smokers in cessation might be relevant for preventing depression. Thus, regardless of the nature of the association between smoking and depression, identifying individuals at risk for both smoking and depression could aid prevention efforts.

It is likely that the association between smoking and depression is at least partly, due to shared liabilities such as genetic and environmental factors common to both smoking and depression (Rose et al. 2009). Based on the genetic correlation of addictive substance use and depression, there are individuals who are genetically vulnerable to both conditions (Brainstorm Consortium et al. 2018). Early onset of smoking has been found to have shared genetic and/or shared environmental liabilities with depression (McCaffery et al. 2008; Silberg et al. 2003). In the scenario of correlated liabilities, it is beneficial to detect those who are genetically vulnerable to both smoking and depressive symptoms as early as possible. On the other hand, if shared environmental factors were found to explain the association, interventions could be targeted, for example, to enhance beneficial family environments as parental substance use and negative parenting practices may increase the risk of smoking and depression (Barman et al. 2004; Mason et al. 2012).

Genetic and other familial factors are, in fact, known to contribute to individual differences in the risk for both smoking and depression (Rice 2009; Rose et al. 2009). Thus, because some familial influences could be shared between smoking and depression, observed associations between the two might reflect familial confounding. A within-pair twin design is highly useful for identifying environmental and genetic confounding through the comparison of monozygotic (MZ) and dizygotic (DZ) twin pairs (McGue et al. 2010). However, studies using genetically informative research designs, including the twin design, are still relatively rare among young populations (Leventhal et al. 2012; McCaffery et al. 2008; Silberg et al. 2003).

Based on the available evidence, interventions for preventing depression in children and adolescents have focused mostly on psychological or psycho-educational programs (Gladstone et al. 2011; Werner-Seidler et al. 2017), but in those programs, substance use has typically not been considered. Using targeted lifestyle strategies such as smoking behavior has been suggested as a possible novel component of population-level prevention initiatives for mental health problems (Jacka et al. 2012). Targeting smoking behavior could prove beneficial since interventions to prevent alcohol misuse have been found effective for preventing psychological and behavioral problems in youth (Castellanos and Conrod 2006). Thus, including substance use prevention to complement psychological interventions could potentially open a new perspective for the prevention of depression.

Apart from confounding by unmeasured shared liabilities, many measured confounders could also account for the observed association. For example, alcohol use has been verified as a confounder for the relationship between smoking and depression (Chaiton et al. 2015). It is crucial to identify and adjust for the potential confounders which can inflate and bias the observed relationship. Such measured confounders associated with both smoking and depression could also be considered as targets of prevention interventions.

Previous studies among adolescents have reported mixed findings regarding the association between smoking and depression and had several limitations. For example, Beal et al. (2014) found that an increase in cigarette smoking predicted an increase in depressive symptoms, but this study only included girls. Further, Gage et al. (2015) found that cigarette smoking predicted increased depressive symptoms, but the evidence was not clear after adjusting for other substance use. Similarly, Albers and Biener (2002) found that rebelliousness accounted for the observed positive correlation between adolescent smoking and later depressive symptoms. In contrast, Audrain-McGovern et al. (2009) reported that smoking progression predicted deceleration of depression symptoms supporting the self-medication hypothesis. Finally, these studies did not utilize genetically informative data and failed to simultaneously address multiple potential confounders, both measured and unmeasured, which could have explained the observed associations.

To address these limitations, this longitudinal study is specifically focused on adolescents and accounts for multiple potential confounders including pre-existing depressiveness. Furthermore, the twin design makes it possible to account for unobserved shared familial influences, such as dispositional genetic or childhood environmental factors. Using prospective data on adolescents from a population-based cohort of twins, our first aim was to examine the predictive association of cigarette smoking at the age of 14 with depressive symptoms at the age of 17 at the individual level. Our second aim was to conduct genetically informative analyses within twin pairs to control for familial confounding by shared genetic and environmental liabilities for smoking and depressive symptoms.

## Method

### Sample

The FinnTwin12 study is a longitudinal population-based twin cohort study initiated in 1994 to examine genetic and environmental factors related to health behaviors and their precursors. It includes Finnish families with twins born in 1983–1987, identified from the Finnish Population Register Centre. A detailed description of the FinnTwin12 study is provided elsewhere (Kaprio et al. 2002; Kaprio 2006).

The first wave of FinnTwin12 began by sending questionnaires to all twins and their parents, starting with a family questionnaire late in the year before the twins reached the age of 12. In the second wave, all participating twins received questionnaires at the age of 14, and the third wave of questionnaire survey was conducted at the age of 17. The age 14 assessment of all twins included a postal questionnaire and had a participation rate of 88.4% with 4740 questionnaires returned out of 5362 mailed. The age 17 follow-up had a participation rate of 92.2% with 4236 questionnaires returned out of 4594 mailed.

In the current study, we used two smoking-related items from the age 14 questionnaire: a number of lifetime cigarettes smoked by the age of 14 (4646 valid answers) and current smoking status at the age of 14 (4691 valid answers). The age 17 questionnaire included an assessment of depressive symptoms using the General Behavior Inventory (GBI) (Depue et al. 1981; Depue 1987) with a total of 4222 responses. For our current analyses, a total of 4110 individuals had non-missing information for both the lifetime cigarettes smoked by the age of 14 and depression scores at the age of 17. Among them, 1361 were monozygotic (MZ) twins (33.1%), 2562 were dizygotic (DZ) twins (same sex 31.4%, opposite sex 31.0%), and 187 were of unknown zygosity. Similarly, 4152 individuals had non-missing information for both the current smoking status at the age of 14 and depression scores at the age of 17. Among them, 1374 were MZ twins (33.1%), 2591 were DZ twins (same sex 31.4%, opposite sex 31.0%), and 187 were of unknown zygosity.

### Measures

#### Depressive Symptoms

The General Behavior Inventory (GBI) is a self-report inventory with items capturing mood-related behaviors, such as depressive, hypomanic, and biphasic symptoms (Depue et al. 1981; Depue 1987). The original scale consists of 73 items, but a shorter version consisting of 10 items was used for the measurement of depressive symptoms (e.g., “Have there been periods of time when you felt a persistent sense of gloom?”). The answers were rated on a 4-point Likert scale from 0 = never to 3 = very often, and we used the total sum score (ranging from 0 to 30) as a continuous count variable. The coefficient alpha was 0.90 for the 10 GBI items, indicating excellent internal consistency of the scale. This shorter version has been successfully used in earlier analyses of data from the FinnTwin12 sample (Edwards et al. 2011; Salmela-Aro et al. 2014).

#### Smoking Behavior

We analyzed two smoking variables at the age of 14: “number of lifetime cigarettes smoked by the age of 14” (cumulative lifetime cigarette exposure) and “current smoking status at the age of 14” (current pattern of smoking at the time of the survey). In the very beginning, individuals were asked: “Have you ever smoked (or tried smoking)?” and the responses were either “yes” or “no.” Those who replied “no” were considered as “never smokers” for both smoking variables. Never smokers thus consisted of those who had never consumed any cigarettes by the age of 14, and this group served as the reference category in the analyses. Those who replied “yes” were invited to answer more detailed smoking-related questions.

To determine the “number of lifetime cigarettes smoked”, participants were asked: “How many cigarettes have you smoked altogether up to now?” and the original responses were “only one,” “about 2–10,” “about 11–50,” and “over 50.” These responses were re-categorized as “1–50 cigarettes” and “more than 50 cigarettes”. The cutoff point of 50 cigarettes has been previously used to indicate regular smoking among youth (Rimpelä et al. 2007).

To determine the “current smoking status”, subjects were asked: “Which of the following best describes your present smoking habits?” and the original responses were “I smoke at least once each day,” “I smoke at least once a week, but not every day,” “I smoke less often than once a week,” “I am trying to or have quit smoking,” and “I have tried smoking but I don’t smoke.” The responses were re-categorized into “experimenters,” “quitters or trying to quit,” and “regular smokers.” Those who reported having smoked but were not smoking during the survey period were considered “experimenters.” Those who reported, at the time of the survey, that they had successfully quit or were trying to quit (currently not smoking) were categorized as “quitters or trying to quit.” “Regular smokers” included participants who smoked cigarettes daily (one or more cigarettes per day) or non-daily (more than once a week but less than daily or less often than once a week). We combined non-daily and daily smokers into “regular smokers” because reporting “non-daily smoking” could also reflect underreporting of smoking frequency in this age group. We found no statistically significant difference in the depression scores between daily and non-daily smokers (results not shown).

#### Covariates

Based on a careful literature review, we considered several variables as potential confounders for the association between smoking and depressive symptoms (Chaiton et al. 2015; Park and Romer 2007). We adjusted our analyses for covariates available at the age of 14, including age, sex, school grades, drinking alcohol to intoxication, health status, and family structure. In addition, we utilized covariates available from a parental questionnaire when the twins were 11–12 years old. These included parental smoking status and education, and pre-existing depressiveness of the twins assessed by their parents.

For determining school grades, the twins were asked: “What kind of grades did you receive last semester compared to the average in your class or course?” and the responses were categorized as “better than average,” “average,” and “below average.” Alcohol use was assessed as drinking alcohol to the point of intoxication. The respondents were asked: “How often do you drink so that you get at least slightly intoxicated?” and the responses were categorized as “once a week or more,” “about 1-2 times a month,” “less than once a month,” and “never, I don’t drink alcohol.” Self-rated health status was measured by asking the respondents: “How do you see your health at the moment?” with responses categorized as “good”, “average,” and “poor.” For determining family structure, respondents were asked: “In addition to you and your siblings, does your family consist of: “a mother and father,” “a mother and stepfather,” “a father and stepmother,” “only a mother,” and “only a father,” and the twins’ families were categorized respectively.

Parental smoking status and education as well as the pre-existing depressiveness of the twins were self-reported and assessed by the parents when the twins were 11–12 years old. Parental smoking was derived from each parent’s questionnaire and was classified as either “both mother and father are non-smokers” or “mother, father, or both are current smokers” (in 37% of participant families, both mother and father were currently smoking). Similarly, for the parents’ highest education level, both mother and father were asked: “What is your basic education?” and classified as “less or equal to intermediate level,” “high school,” or “university degree.” Pre-existing depressiveness was assessed by the parents using the parent version of the multidimensional peer nomination inventory (MPNI) (Pulkkinen et al. 1999). The MPNI covers a wide spectrum of externalizing, internalizing, and prosocial behaviors, and includes five items for assessing depressiveness. These five items were “sad or depressed a lot of the time,” “easily hurt if others are mean to him/her,” “lonely and has no friends,” “often worried,” and “clings to adults or is too dependent on them.” Each of the five items was rated on a 0–3 scale. A mean score was calculated and used as a single continuous variable.

### Statistical Analyses

In the first set of analyses, we considered twins as individuals. Because observations within twin pairs are correlated, the non-independence owing to twinship was statistically accounted for by using a robust variance estimator (Williams 2000). The association between smoking and subsequent depressive symptoms was analyzed with negative binomial regression. This was the most appropriate model because of the positively skewed outcome variable (floor effect), which had an unequal mean and variance. We calculated incidence rate ratios (IRR) with 95% confidence intervals (CI) and considered IRRs with *p* < 0.05 statistically significant.

The negative binomial regression models were first adjusted for age and sex. Next, to this crude model, we included the covariates: school grades, drinking alcohol to intoxication, health status, family structure, parental education, and parental smoking status simultaneously. In the final model, we adjusted for all the covariates plus pre-existing depressiveness. In addition to these three main models, we assessed the impact of each covariate by adding the covariates individually to the crude model. We also tested for a sex-by-smoking interaction on depressive symptoms. Data for males and females were pooled together in the analyses because there were no statistically significant interactions for either smoking variable: lifetime cigarettes smoked (*p* = 0.31) and current smoking status at the age of 14 (*p* = 0.62).

To control for possible confounding due to genetic and other familial influences, we conducted within twin pair analyses using fixed-effects negative binomial regression (Allison 2009). These analyses were stratified by twin pair and, thus, by design adjusted for all unmeasured factors which are constant within pairs, including shared genetic and environmental factors. The within-pair analysis included first the crude model and then the fully adjusted model which included all covariates simultaneously. This analysis was first conducted among all pairs and then separately among MZ and DZ pairs (same-sex and opposite-sex pairs pooled together and adjusted for sex).

In both the individual and within-pair analyses, the number of observations in the fully adjusted model was smaller than in the other models. Thus, we tested whether the loss of observations could have affected our results by comparing the age- and sex-adjusted estimates among those with no missing values to the estimates in the full sample. As another sensitivity analysis, we also investigated the reverse association between depressive symptoms, assessed at ages 11–12, and smoking at the age of 17. We used multinomial logistic regression to calculate relative risk ratios (RRR) with 95% CIs and considered RRRs with *p* < 0.05 statistically significant. The analysis was adjusted for all the same covariates as the main analyses. Furthermore, we also conducted a drop-out analysis to observe if current smoking at the age of 14 predicted dropout at the age 17 survey. All the analyses were conducted using the Stata statistical software (version 13) (StataCorp 2013).

## Results

### Descriptive Statistics

By the age of 14, one-third of the participants had smoked 1–50 cigarettes and 8% had smoked more than 50 cigarettes (Table 1). More than one-fourth of the twins were experimenters, 4% had quit or were trying to quit, and 9% were regular smokers. There was a statistically significant sex difference regarding current smoking status at the age of 14 (*χ*^2^ (3) = 20.1, *p* < 0.001), more specifically, there were more experimenters among males, whereas more quitters or those trying to quit and regular smokers among females. Females reported higher depression scores than males (Mann-Whitney *U* = 18.2, *p* < 0.001) (Table 1; Supplementary Figs. 1a, b).

**Table 1:** Descriptive statistics of cigarette smoking at the age of 14 and depression scores at the age of 17 by sex

Lifetime cigarettes smoked *N* (%)	Total (*N* = 4646)	Male (*N* = 2316)	Female (*N* = 2330)
None	2696 (58.0%)	1337 (57.7%)	1359 (58.3%)
1–50	1579 (34.0%)	796 (34.4%)	783 (33.6%)
> 50	371 (8.0%)	183 (7.9%)	188 (8.1%)
*χ*^2^ = 0.31, df = 2, *p* = 0.856			
Current smoking status at the age of 14 *N* (%)	Total (*N* = 4691)	Male (*N* = 2333)	Female (*N* = 2358)
Never smokers	2696 (57.5%)	1337 (57.3%)	1359 (57.6%)
Experimenters	1370 (29.2%)	729 (31.2%)	641 (27.2%)
Quitters or trying to quit	196 (4.2%)	90 (3.9%)	106 (4.5%)
Regular smokers	429 (9.1%)	177 (7.6%)	252 (10.7%)
*χ*^2^ = 20.1, df = 3, *p* < 0.001			
Depression score	Total (*N* = 4222)	Male (*N* = 2036)	Female (*N* = 2186)
Mean (SD)	5.1 (4.9)	3.7 (4.0)	6.3 (5.4)
Median (Q1, Q3)	4 (1.7)	3 (1.5)	5 (2.9)
Skewness	1.66	1.88	1.45
Sum score 0	13.4%	18.7%	8.4%
Sum score 1–10	75.4%	75.2%	75.6%
Sum score 11–20	9.6%	5.6%	13.4%
Sum score 21–30	1.6%	0.5%	2.6%
Mann-Whitney *U* test: *z* = 18.2, *p* < 0.001			

*N*, total number; *SD*, standard deviation; Q1, 1st quartile; Q3, 3rd quartile

### Association between Smoking at the Age of 14 and Depressive Symptoms at the Age of 17

#### Lifetime Cigarettes Smoked

A higher number of cigarettes smoked by the age of 14 were associated with higher depression scores at the age of 17 (Table 2). Compared to never smokers, depression scores were 19% higher among those who had smoked 1–50 cigarettes and 43% higher among those who had smoked more than 50 cigarettes when adjusted for age and sex. The association was attenuated but remained statistically significant when adjusted for each covariate individually (e.g., estimate for having smoked > 50 cigarettes, adjusting for drinking to intoxication, IRR = 1.22, 95% CI 1.06–1.40) (Supplementary Table 1), and when adjusted for all covariates and pre-existing depressiveness simultaneously (Table 2).

**Table 2:** Negative binomial regression analysis for the depression score outcome (at the age of 17) by smoking behavior (at the age of 14)

Cigarette smoking^a^	Adjusted for age and sex			Adjusted for all covariates			Adjusted for all covariates and pre-existing depressiveness		
	IRR	95% CI	*p*	IRR	95% CI	*p*	IRR	95% CI	*p*
Lifetime cigarettes smoked	*N* = 4110			*N* = 4110			*N* = 3923		
1–50	1.19	1.11–1.27	2.5e-07	1.09	1.01–1.17	0.021	1.08	1.01–1.17	0.032
> 50	1.43	1.28–1.60	7.1e-10	1.12	0.97–1.29	0.121	1.17	1.01–1.35	0.037
Current smoking status at the age of 14	*N* = 4152			*N* = 4152			*N* = 3960		
Experimenters	1.16	1.08–1.25	2.2e-05	1.10	1.02–1.18	0.012	1.09	1.01–1.18	0.021
Quitters or trying to quit	1.31	1.14–1.51	1.8e-04	1.12	0.96–1.29	0.143	1.11	0.96–1.29	0.160
Regular smokers	1.46	1.32–1.62	8.1e-14	1.16	1.01–1.32	0.031	1.19	1.03–1.36	0.015

^a^Reference category: never smokers

IRR, incidence rate ratio; CI, confidence interval

All covariates: age, sex, school grades, drinking alcohol to intoxication, health status, family structure, parental smoking status, and parental education

#### Current Smoking Status at the Age of 14

Likewise, current smoking status at the age of 14 was associated with higher depression scores at the age of 17 (Table 2). When adjusted for age and sex, and using never smokers as the reference group, depression scores were 16% and 31% higher in experimenters and quitters or those trying to quit, respectively, and 46% higher in regular smokers. There was clear evidence for the association after adjusting for each covariate individually (Supplementary Table 1). After adjusting for all covariates and pre-existing depressiveness simultaneously, the associations were attenuated but remained evident among experimenters (IRR = 1.09, 95% CI 1.01–1.18) and regular smokers (IRR = 1.19, 95% CI 1.03–1.36) (Table 2).

### Within-Pair Analysis

In within-pair analyses including both zygosities, smoking was associated with depression scores, and the IRR was highest for regular smokers (IRR = 1.23, 95% CI 1.08–1.40) and quitters or those trying to quit (IRR = 1.22, 95% CI 1.05–1.41) (Table 3). In the fully adjusted model, the estimates were somewhat attenuated. In zygosity-specific analyses, a significant association between smoking and depressive symptoms was found in DZ pairs but not within MZ pairs. Within DZ pairs, being a regular smoker was associated with 26% higher depression scores compared to never smokers. Similarly, being a quitter or trying to quit was associated with 25% higher depression scores. The estimates were attenuated in the fully adjusted models (Table 3).

**Table 3:** Within-pair fixed-effects negative binomial regression models for the depression score outcome (at the age of 17) by smoking behavior (at the age of 14)

Cigarette smoking^a^	All pairs						DZ						MZ					
	Crude model^b^			Adjusted model			Crude model^b^			Adjusted model			Crude model			Adjusted model		
	IRR	95% CI	*p*	IRR	95% CI	*p*	IRR	95% CI	*p*	IRR	95% CI	*p*	IRR	95% CI	*p*	IRR	95% CI	*p*
Lifetime cigarettes smoked	*N* = 1877 pairs			*N* = 1789 pairs			*N* = 1176 pairs			*N* = 1120 pairs			*N* = 614 pairs			*N* = 585 pairs		
1–50	1.10	1.02–1.19	0.018	1.07	0.98–1.16	0.114	1.12	1.01–1.24	0.025	1.09	0.98–1.21	0.101	1.01	0.83–1.22	0.935	1.00	0.83–1.21	0.971
> 50	1.13	0.99–1.30	0.069	1.05	0.90–1.22	0.521	1.14	0.96–1.36	0.139	1.06	0.87–1.28	0.571	1.11	0.86–1.44	0.413	1.08	0.80–1.44	0.625
Current smoking status at the age of 14	*N* = 1910 pairs			*N* = 1819 pairs			*N* = 1199 pairs			*N* = 1140 pairs			*N* = 624 pairs			*N* = 595 pairs		
Experimenters	1.09	1.00–1.18	0.041	1.06	0.98–1.15	0.155	1.10	1.00–1.21	0.056	1.08	0.97–1.20	0.143	1.03	0.86–1.24	0.719	1.04	0.87–1.24	0.655
Quitters or trying to quit	1.22	1.05–1.41	0.007	1.14	0.97–1.34	0.109	1.25	1.04–1.51	0.020	1.17	0.96–1.44	0.126	1.07	0.82–1.39	0.625	1.07	0.82–1.41	0.610
Regular smokers	1.23	1.08–1.40	0.002	1.10	0.95–1.27	0.205	1.26	1.07–1.48	0.006	1.12	0.93–1.35	0.219	1.09	0.85–1.40	0.481	1.08	0.82–1.42	0.581

^a^Reference category: never smokers

^b^Adjusted for sex in all pairs and DZ pairs

IRR, incidence rate ratio; *CI*, confidence interval

Adjusted model: sex, school grades, drinking alcohol to intoxication, health status, and pre-existing depressiveness

### Sensitivity Analyses

As a sensitivity analysis, we checked whether the listwise exclusion of observations with missing values in the covariates might have biased our results. We found no support for such a bias as analyses using only observations with no missing data provided nearly identical estimates as those in the full dataset (results not shown). We also conducted a drop-out analysis and found that current smoking at the age of 14 significantly predicted dropout from the age 17 survey (OR = 1.66, 95% CI 1.20–2.30), especially among those who were daily smokers at the age of 14 (OR = 2.55, 95% CI 1.73–3.76). As another sensitivity analysis, we checked for the reverse association (i.e., whether depressive symptoms assessed at ages 11–12 predicted smoking at the age of 17). Depressive symptoms were not associated with smoking at follow-up (results not shown).

## Discussion

In this longitudinal study of adolescent twins, we found that compared to never smokers, regular smokers at the age of 14, and those having consumed a higher number of cigarettes by that age had higher depression scores at the age of 17. The result is in line with earlier longitudinal studies focusing on adolescents (Chaiton et al. 2009). However, our study included an adjustment for several measured confounders as well as within twin-pair comparisons to control for shared familial liabilities. Our findings suggest that the observed associations between smoking and subsequent depressive symptoms are confounded by a combination of several measured covariates and by unmeasured familial factors, mainly by shared genetic factors rather than shared childhood environment.

The descriptive findings of our study indicate that a significant percentage of adolescents experiment with cigarette smoking by the age of 14, and many of them are reportedly regular smokers. Furthermore, female participants had a slightly higher prevalence of smoking and higher depression scores compared to males. Our study found a lower prevalence of adolescent smokers compared to national statistics in Finland, where 14% of boys and 17% of girls among 14 years old were already daily smokers in 1997 (Kinnunen et al. 2015), the year when data collection for the age of 14 measurements was initiated for the FinnTwin12 study. This difference may be due to biased dropout in our follow-up study. Based on our analyses of missing data, it was evident that those who were daily smokers at the age of 14 were more likely to drop out from the follow-up survey at the age of 17. Additionally, our twins were assessed immediately after their 14th birthday, so they are few months younger compared to the national sample.

As expected, we observed a predictive association of smoking with depressive symptoms such that regular smokers had higher depression scores compared to never smokers. Similar results were found in previous studies where adolescents who were daily smokers, or those having higher levels of smoking, were more likely to report depressive symptoms later (Beal et al. 2014; Goodman and Capitman 2000; Wilkinson et al. 2016). In contrast, Audrain-McGovern et al. (2009) found that higher levels of smoking predicted a decrease in depressive symptoms, whereas higher depressive symptoms predicted an increase in smoking, mediated through an increase in the number of smoking peers. Our results among quitters or those trying to quit, after adjusting for covariates, demonstrated no significant association. This may be due to the heterogeneous nature of this group of respondents, who consist of some who have already quit smoking and others still in the process of quitting at the time of the survey. Interestingly, in our results, also experimenters had an increased risk for depressive symptoms. This may be because some symptoms of nicotine dependence are already present even among adolescents who are experimenters and hence, they can suffer from withdrawal symptoms, such as depressed mood, on days when they do not smoke (DiFranza 2015). Although those smoking less (e.g., experimenters) may have an increased risk for depressive symptoms, in the long term, smoking cessation seems to be associated with reduced depression, as well as improved positive mood and quality of life (Taylor et al. 2014b).

To account for the potential confounding effect of genetic and other familial factors, we conducted within-pair analyses. Conducting such analyses in MZ and DZ twin pairs helps to understand the familial liabilities shared by smoking and depression. In the within twin-pair analysis, typically, there are three possible scenarios. First, if the observed association is similar when studying twins as individuals and in within twin-pair analysis (where co-twins differ both in the exposure and the outcome) within both MZ and DZ pairs, there is no evidence for genetic or shared environmental confounding, and the association may be compatible with a causal interpretation. Second, if the observed association is similar within MZ and DZ pairs but is clearly reduced compared to the population, this suggests the association is confounded by shared environmental factors which are equally shared between MZ and DZ co-twins. Third, if the association is smaller within MZ pairs compared to DZ pairs which also show a reduced association compared to the population, this suggests the association is confounded by shared genetic factors. Comparison between MZ and DZ pairs allows to differentiate the origin of the confounding (shared genetic or shared environmental factors) because co-twins from both MZ and DZ pairs raised together share 100% of their childhood family environment, whereas co-twins from DZ pairs share on average 50% and those from MZ pairs 100% of genetic variance. Thus, in our study, the significant association seen within DZ pairs has likely occurred due to genetic factors which the co-twins do not share. This illustrates the concept of genetic confounding, i.e., a situation where an association between two variables arises due to shared genetic influences and is gradually reduced when more genetic covariance is accounted for, first by comparing DZ co-twins and then MZ co-twins. In our study, there was clear support for genetic confounding, as the population level association between regular smoking at the age of 14 and depressive symptoms at the age of 17 (age and sex adjusted, IRR = 1.46, 95% CI 1.32–1.62) was gradually attenuated in the within-pair analysis (DZ pairs, IRR = 1.26, 95% CI 1.07–1.48; MZ pairs, IRR = 1.09, 95% CI 0.85–1.40). It should be noted that while the smaller sample size in MZ pairs resulted in limited statistical power, point estimates in MZ pairs were in most cases close to unity, suggesting no association.

Our results are supported by a study among adolescents, where shared genetic factors were partly responsible for the correlation between cigarette smoking and depression in females (McCaffery et al. 2008). Similarly, in another study, the correlation between cigarette smoking and depression was explained by shared liabilities, more specifically, by genetic influences in females and by environmental influences in males (Silberg et al. 2003). However, in a previous Finnish study using a discordant twin pair design among adult twins, risk estimates for daily smokers were clearly elevated in the individual-based analysis, as well as in the within-discordant-MZ-and-DZ-pair analysis (although statistically not significant), suggesting the association was not completely explained by shared familial factors (Korhonen et al. 2017). Depression was measured in that study by a population register-based outcome (use of antidepressants), likely denoting more severe depression and possibly of greater duration given the diagnosed symptoms, and exposure to smoking was more extreme, such as heavy daily smoking. Interestingly, a study using the Mendelian randomization approach in adults did not find support for a causal role of cigarette smoking in the development of depression (Taylor et al. 2014a). Thus, taken together from the previous literature, the causal role of smoking in the development of depression remains uncertain. Our study findings observed in an adolescent sample support the hypothesis that the observed association may be confounded by shared genetic factors but offers no support for a causal effect of smoking on depressive symptoms.

We also considered several measured confounders, including age, sex, school grades, drinking alcohol to intoxication, health status, family structure, parental smoking status, parental education, and pre-existing depressiveness, many of which have been suggested as potential confounders for the association between smoking and depression (Chaiton et al. 2015; Park and Romer 2007). The association between cigarette use and depression can be attenuated after adjusting for multiple confounders including alcohol use (Gage et al. 2015). Also, in our study, the magnitude of the associations of interest decreased notably when we accounted for drinking alcohol to intoxication. The explanation for this result is that individuals consuming alcohol to intoxication are also more likely smokers; furthermore, excess alcohol use is a well-established risk factor for depressive symptoms (Pedrelli et al. 2016). The relationship of smoking with alcohol use is complex, and it is unclear which substance is driving the association with depression.

Although shared genetic factors and measured confounders appeared to account for the association between smoking and depressive symptoms in our study, identifying individuals who are vulnerable to both depression and smoking would be beneficial for the prevention of depression and smoking initiation. Interventions can be targeted towards the prevention of both smoking and depression simultaneously (Mason et al. 2012). In fact, smoking prevention programs have incorporated cognitive behavior and life-skill modalities (Hwang et al. 2004), which may be relevant in depression prevention, for example, by incorporating substance-use minimization. Such preventive aspects should target the young population, who are prone to risky behaviors including substance abuse and mental health problems.

We utilized two smoking variables—cumulative lifetime cigarette exposure and current pattern of smoking at the time of the survey. The total number of cigarettes smoked during lifetime represents the very early stages of smoking behavior, and progression to having smoked more than 50 cigarettes could indicate increased tolerance. On the other hand, the self-attribution to be a daily, weekly, or less than a weekly smoker, and considering oneself as only an experimenter or already trying to quit, is an assessment of the self-image of the respondent as a smoker, which is based on the actual smoking behavior. These smoking variables can be seen to represent quantitative and qualitative dimensions in smoking measurement. Therefore, although, the cumulative amount and the self-attribution of current smoking status are correlated, they also capture partly different aspects of smoking behavior.

Although the focus of this study was to understand if smoking at the age of 14 predicts depressive symptoms at the age of 17, we also conducted a sensitivity analysis to investigate if earlier depressive symptoms predict smoking at the age of 17. Such a bidirectional association between depression and smoking has been found among adolescents (Chaiton et al. 2009). In our study, depressiveness assessed by the parents of the twin at ages 11–12 did not predict smoking during follow-up. Earlier studies have provided conflicting evidence on whether depressive symptoms are a risk factor for the development of smoking behavior (Audrain-McGovern et al. 2009; Beal et al. 2014; Goodman and Capitman 2000; Pedersen and von Soest 2009; Wilkinson et al. 2016).

Our study is based on a unique population-based longitudinal dataset of twins followed up for a 3-year period from the age of 14 to age of 17, covering an important phase of substance use initiation and emerging mental health problems. Another strength of this study is the available large sample size and high participation rates. Next, we were also able to adjust for important confounders such as pre-existing depressive symptoms, parental education, and parental smoking behavior. Most importantly, analyses within twin pairs controlling for shared familial and genetic influences provided more robust estimates of the association. That estimates were lower within MZ pairs (close to unity) compared to DZ pairs indicated that the likely source of confounding was due to shared genetic rather than shared environmental factors. Additionally, we studied the reverse association from depressiveness to smoking but found no evidence for depressive symptoms increasing the risk of becoming a smoker.

One limitation of our study was that we used self-reported data and our measure of depression was non-diagnostic. However, the 10-item GBI depressive score has provided meaningful results in previous studies (Edwards et al. 2011; Salmela-Aro et al. 2014), and the GBI demonstrated high internal consistency in our sample. Second, there were relatively few regular smokers in our sample (9%) limiting the statistical power in our analyses. This is due to regular smoking being rare in these younger age groups. Third, the findings of the within-pair analyses were not always completely clear as some point estimates were relatively similar in MZ and DZ pairs. Also, the former smoker category not reaching statistical significance may have been due to limited statistical power. Further, adjusting for pre-existing depressive symptoms resulted in some observations being lost because of missing data. However, sensitivity analyses among those with non-missing data provided similar results as the full sample.

In conclusion, cigarette smoking during early adolescence is associated with reporting more depressive symptoms later in adolescence but this association is likely to arise because of confounding due to measured factors as well as underlying genetic influences. However, even though no support for a causal association was found, information on smoking in adolescence can be useful for prevention efforts as a proxy for elevated risk for depression.

## Electronic Supplementary Material

ESM 1 (DOCX 28.3 kb)

ESM 2 (DOCX 23 kb)
