# Supplementary material for: Predictive Association of Smoking with Depressive Symptoms: a Longitudinal Study of Adolescent Twins
Source: Prev Sci. 2019 May 8;20(7):1021–30. doi: 10.1007/s11121-019-01020-6 (PMC6718365; doi:10.1007/s11121-019-01020-6)
Supplement: Supplementary file 1 — (DOCX 28.3 kb) [file 11121_2019_1020_MOESM1_ESM.docx]

| **Cigarette smoking** ^a^ | **Crude Model** | | | **Adjusted for age and sex** | | | **Adjusted for age, sex and grades** | | | **Adjusted for age, sex and drinking alcohol to intoxication** | | | **Adjusted for age, sex**  **and health status** | | |
| --- | --- | --- | --- | --- | --- | --- | --- | --- | --- | --- | --- | --- | --- | --- | --- |
|  | **IRR** | **95% CI** | ***p*** | **IRR** | **95% CI** | ***p*** | **IRR** | **95% CI** | ***p*** | **IRR** | **95% CI** | ***p*** | **IRR** | **95% CI** | ***p*** |
| **Lifetime cigarettes smoked** | **N=4,110** | | | **N=4,110** | | | **N=4,110** | | | **N=4,110** | | | **N=4,110** | | |
| 1-50 | 1.19 | 1.11- 1.28 | 7.1e-07 | 1.19 | 1.11-1.27 | 2.5e-07 | 1.18 | 1.10-1.26 | 1.0e-06 | 1.11 | 1.03-1.20 | 0.005 | 1.18 | 1.10-1.26 | 1.5e-06 |
| > 50 | 1.48 | 1.32-1.67 | 9.8e-11 | 1.43 | 1.28-1.60 | 7.1e-10 | 1.39 | 1.24-1.56 | 3.4e-08 | 1.22 | 1.06-1.40 | 0.007 | 1.34 | 1.19-1.50 | 8.0e-07 |
| **Current smoking status at the age of 14** | **N=4,152** | | | **N=4,152** | | | **N=4,152** | | | **N=4,152** | | | **N=4,152** | | |
| Experimenters | 1.15 | 1.07-1.24 | 1.8e-04 | 1.16 | 1.08-1.25 | 2.2e-05 | 1.16 | 1.08-1.24 | 5.0e-05 | 1.11 | 1.03-1.20 | 0.004 | 1.16 | 1.08-1.24 | 4.1e-05 |
| Quitters or trying to quit | 1.34 | 1.15-1.56 | 1.4e-04 | 1.31 | 1.14-1.51 | 1.8e-04 | 1.28 | 1.11-1.48 | 0.001 | 1.19 | 1.03-1.38 | 0.018 | 1.25 | 1.09-1.44 | 0.002 |
| Regular smokers | 1.56 | 1.40-1.72 | 2.1e-17 | 1.46 | 1.32-1.62 | 8.1e-14 | 1.43 | 1.29-1.59 | 7.3e-12 | 1.26 | 1.10-1.44 | 0.001 | 1.38 | 1.24-1.53 | 7.3e-10 |

**Supplementary Table 1 Negative binomial regression analysis for the depression score outcome (at the age of 17) by smoking behavior (at the age of 14): Results from multiple models where each covariate has been added individually**

(continued)

**Supplementary Table 1 (Continued)**

| **Cigarette smoking** ^a^ | **Adjusted for age, sex and family structure** | | | **Adjusted for age, sex**  **and parental smoking** | | | **Adjusted for age, sex**  **and parental education** | | | **Adjusted for age, sex and pre-existing**  **depressiveness** | | | **Adjusted for all covariates and pre-existing**  **depressiveness** | | |
| --- | --- | --- | --- | --- | --- | --- | --- | --- | --- | --- | --- | --- | --- | --- | --- |
|  | **IRR** | **95% CI** | ***p*** | **IRR** | **95% CI** | ***p*** | **IRR** | **95% CI** | ***p*** | **IRR** | **95% CI** | ***p*** | **IRR** | **95% CI** | ***p*** |
| **Lifetime cigarettes smoked** | **N=4,100** | | | **N=4,100** | | | **N=4,100** | | | **N=** **3,923** | | | **N=3,923** | | |
| 1-50 | 1.18 | 1.11-1.27 | 5.5e-07 | 1.18 | 1.10-1.26 | 1.4e-06 | 1.19 | 1.11-1.27 | 3.4e-07 | 1.18 | 1.10-1.26 | 2.3e-06 | 1.08 | 1.01-1.17 | 0.032 |
| >50 | 1.40 | 1.25-1.57 | 7.0e-09 | 1.41 | 1.26-1.58 | 3.5e-09 | 1.42 | 1.27-1.59 | 9.3e-10 | 1.48 | 1.31-1.66 | 1.1e-10 | 1.17 | 1.01-1.35 | 0.037 |
| **Current smoking status at the age of 14** | **N=4,152** | | | **N=4,152** | | | **N=4,152** | | | **N=** **3,960** | | | **N=3,960** | | |
| Experimenters | 1.16 | 1.08-1.24 | 3.3e-05 | 1.16 | 1.08-1.24 | 5.6e-05 | 1.16 | 1.08-1.25 | 2.3e-05 | 1.15 | 1.07-1.23 | 1.1e-04 | 1.09 | 1.01-1.18 | 0.021 |
| Quitters or trying to quit | 1.30 | 1.13-1.50 | 2.9e-04 | 1.29 | 1.12-1.49 | 4.3e-04 | 1.30 | 1.13-1.50 | 2.3e-04 | 1.29 | 1.12-1.49 | 5.5e-04 | 1.11 | 0.96-1.29 | 0.160 |
| Regular smokers | 1.44 | 1.30-1.59 | 6.5e-13 | 1.45 | 1.31-1.60 | 6.0e-13 | 1.45 | 1.32-1.61 | 2.0e-13 | 1.49 | 1.34-1.65 | 6.2e-14 | 1.19 | 1.03-1.36 | 0.015 |

^a^ Reference category: Never Smokers

IRR = Incidence Rate Ratio, CI = Confidence Interval

All covariates = age, sex, school grades, drinking alcohol to intoxication, health status, family structure, parental smoking status and parental education
