# Supplementary material for: Predictive Association of Smoking with Depressive Symptoms: a Longitudinal Study of Adolescent Twins
Source: Prev Sci. 2019 May 8;20(7):1021–30. doi: 10.1007/s11121-019-01020-6 (PMC6718365; doi:10.1007/s11121-019-01020-6)
Supplement: Supplementary file 2 — (DOCX 23 kb) [file 11121_2019_1020_MOESM2_ESM.docx]

Supplementary Figure 1a. Box plot of depression score by lifetime cigarettes smoked and sex.

 Supplementary Figure 1b. Box plot of depression score by current smoking status at age 14 and sex.
